# The analgesic effect and neural mechanism of spicy food intake

**DOI:** 10.1093/scan/nsaf040

**Published:** 2025-05-02

**Authors:** Bojun He, Min Shao, Junyu Wu, Junyao Wang, Zilong Wei, Lu Chen, Jing Meng

**Affiliations:** Research Center for Brain and Cognitive Science, School of Educational Sciences, Chongqing Normal University, Chongqing 401331, China; Key Laboratory of Applied Psychology, School of Educational Sciences, Chongqing Normal University, Chongqing 401331, China; Research Center for Brain and Cognitive Science, School of Educational Sciences, Chongqing Normal University, Chongqing 401331, China; Key Laboratory of Applied Psychology, School of Educational Sciences, Chongqing Normal University, Chongqing 401331, China; Research Center for Brain and Cognitive Science, School of Educational Sciences, Chongqing Normal University, Chongqing 401331, China; Key Laboratory of Applied Psychology, School of Educational Sciences, Chongqing Normal University, Chongqing 401331, China; Research Center for Brain and Cognitive Science, School of Educational Sciences, Chongqing Normal University, Chongqing 401331, China; Key Laboratory of Applied Psychology, School of Educational Sciences, Chongqing Normal University, Chongqing 401331, China; Research Center for Brain and Cognitive Science, School of Educational Sciences, Chongqing Normal University, Chongqing 401331, China; Key Laboratory of Applied Psychology, School of Educational Sciences, Chongqing Normal University, Chongqing 401331, China; Research Center for Brain and Cognitive Science, School of Educational Sciences, Chongqing Normal University, Chongqing 401331, China; Key Laboratory of Applied Psychology, School of Educational Sciences, Chongqing Normal University, Chongqing 401331, China; Research Center for Brain and Cognitive Science, School of Educational Sciences, Chongqing Normal University, Chongqing 401331, China; Key Laboratory of Applied Psychology, School of Educational Sciences, Chongqing Normal University, Chongqing 401331, China

**Keywords:** pain, food, analgesia, spicy, event-related potential

## Abstract

Although published studies have shown that applying capsaicin to the skin can have an analgesic effect on other parts of the body, the impact of spicy food intake on pain perception and its neurological mechanism remains unclear. Thus, two studies utilizing questionnaires and experiments with event-related potential (ERP) technology were conducted to explore this question. Study 1 recruited 300 adults and found a negative correlation between spicy food cravings and pain perception in daily life. Study 2 involved 45 participants and examined behavioural and ERP responses to pain (including minor pain and moderate pain) stimuli following spicy and control treatments. Results showed that, compared to control treatments, spicy treatments led to shorter reaction times, lower accuracies and pain intensity ratings, less negative emotional responses, smaller N1 and P2 amplitudes, and shorter N1 and P2 latencies, especially for minor-pain stimuli. These findings indicate that spicy food intake may have an analgesic effect.

## Introduction

Spicy food, particularly chili peppers, enjoys widespread and increasing global popularity (Choy et al. 2021, Kwon et al. 2021). Capsaicin, the primary pungent component in chili, is known to stimulate pain receptors in humans (Nilius and Appendino 2013, Duan et al. 2020), triggering a burning pain sensation on the skin and tongue (Yuling et al. 2018, Li et al. 2020, 2022, Alexandre et al. 2024). Interestingly, applying capsaicin to the skin has been shown to have an analgesic effect on other parts of the body (Derry et al. 2017). Hence, we are curious whether spicy sensation could suppress the perception of pain stimuli and its underlying neurological mechanism.

Nowadays, pain is a common clinical condition and a widespread global health concern (Goldberg and McGee 2011, Li and Hu 2016, Lin et al. 2022). While medication is effective for treating pain, challenges such as drug addiction and side effects pose significant hurdles to pain relief (Staahl et al. 2009, Rütgen et al. 2018). Fortunately, nonpharmacological analgesic interventions, including dietary therapy (Wiwanitkit 2010, Arora et al. 2021, 2022, Feng et al. 2022), have shown promising effects in recent years.

Event-related potential (ERP) components related to pain, known as N1 and P2, typically emerge ∼100 and 200 ms following a pain stimulus. N1 represents the early component of pain interpretation and sensory information processing (Lee et al. 2009, Mouraux and Iannetti 2009, Peng et al. 2019, Zhang et al. 2023), while P2 represents a subsequent mental processing stage concerning the affective-motivational component of pain (Rütgen et al. 2015, Li et al. 2023, Khondakar et al. 2024).

One behavioural study preliminarily explored the relationship between spicy food stimulation and pain threshold. It measured participants’ pressure and cold pain thresholds after treating their tongue with either a spicy solution or pure water. The study found that the spicy treatment increased the pain threshold compared to the water treatment (Duan et al. 2020). Additionally, it examined the correlation between participants’ daily spicy food consumption frequency and their pain thresholds, finding a negative association between spicy diet frequency and pain thresholds (Duan et al. 2020). However, this study did not investigate the effect of spicy food intake on pain stimuli beyond the pain threshold or explore the relationship between daily spicy cravings and perception of higher-intensity pain. Hence, the main aim of this study was to investigate the effect of actual oral spicy food intake on the perception of both minor pain and moderate pain.

To better understand the potential cognitive and neural mechanisms of spicy food intake on pain perception, we conducted the following two studies. Study 1 investigated the correlation between individuals’ spicy food craving and pain perception using a questionnaire method. Study 2 explored the effect of spicy food intake on pain perception using the ERP method. Based on previous studies (Duan et al. 2020, Yang et al. 2023), we hypothesized that individuals who prefer spicy food would exhibit decreased pain perception, and that spicy food intake might reduce pain perception and result in decreased N1 and P2 amplitudes.

## Materials and methods

### Study 1: The relationship between spicy food craving and pain perception

#### Participants

The required sample size for the correlation analysis was calculated using G*Power 3. Assuming a medium effect size (*r* = 0.3), a two-tailed test, an alpha level of 0.05, and a power of 0.95, the calculation indicated that a minimum sample size of 138 participants would be required. A total of 300 adults (189 females) aged 18 and 23 years (mean = 19.51 years, SD = 0.87 years) from Chongqing Normal University, Chongqing, China, were recruited for this study. All participants had normal or corrected-to-normal vision, had no neurological or psychiatric disorders, had no acute or chronic pain, and were not currently using any medication. Written informed consent was obtained from all participants prior to participation. This study conformed to all provisions of the Declaration of Helsinki and was approved by the local research ethics committee of Chongqing Normal University. All procedures followed ethical guidelines and regulations.

#### Measures

##### Spicy Food Craving

The Mandarin version (Zhou et al. 2019) of the Spicy Food Craving Questionnaire (Meule and Hormes 2015), a reliable tool for measuring individual spicy food craving, was used in this study. The questionnaire consists of 12 self-report items and two polygraph questions (e.g. ‘I have never had spicy food before.’) across two subscales: behaviour and positive reinforcement, cognition and thoughts. Seven items (1, 3, 4, 5, 8, 10, and 14) contribute to the behaviour and positive reinforcement subscale (e.g. ‘I eat spicy food every day.’), while five items (2, 6, 9, 12, and 13) contribute to the cognition and thoughts subscale (e.g. ‘When I crave spicy food, I want to eat it right away.’). Participants evaluated each item on a seven-point scale (1* =* ‘not at all’, 7 = ‘completely’). Higher scores represent higher levels of spicy food craving. In this study, Cronbach’s *α* of the Spicy Food Craving Questionnaire was .94.

##### Pain Perception

The Mandarin version (Quan et al. 2018) of the Pain Sensitivity Questionnaire (Ruscheweyh et al. 2009) has proven to be a valid instrument for evaluating pain perception among healthy adults (Ruscheweyh et al. 2009). The Pain Sensitivity Questionnaire comprises 17 self-report items and measures pain perception across two subscales: minor and moderate pain. Seven items (3, 6, 7, 10, 11, 12, and 14) contribute to the minor-pain subscale (e.g. ‘Imagine a mild sunburn on your shoulders.’), and seven items (1, 2, 4, 8, 15, 16, and 17) contribute to the moderate-pain subscale (e.g. ‘Imagine you trap your finger in a drawer.’). Three items (5, 9, and 13) describe normally nonpainful situations (e.g. ‘Imagine you take a shower with lukewarm water.’). Participants rated how painful these situations would be for them on a 10-point Likert scale (0 = ‘not painful at all’, 10 = ‘worst pain imaginable’). The Pain Sensitivity Questionnaire scores were calculated with an average of 14 items from the minor-pain and moderate-pain subscales. Higher scores represent higher pain perception. In this study, Cronbach’s *α* of the Pain Sensitivity Questionnaire was .90.

#### Data analysis

Data analyses were performed using MATLAB R2016a (MathWorks, Natick, MA, USA), including descriptive statistics, internal reliability (*α*) estimates, and correlation analyses. Pearson’s product-moment correlation analysis was used to examine the relationship between Spicy Food Craving and Pain Perception. Tests of normality showed no significant deviation from normality for the study variables (e.g. skewness < |3.0| and kurtosis < |10.0|) (Drezner et al. 2010, Kline 2018). The *P*-values were corrected using Bonferroni method to account for the multiple comparison problems.

### Study 2: The influence of spicy food intake on pain perception

#### Participants

Participants were recruited through posters and online advertising at Chongqing Normal University, Chongqing, China. *A priori* power analysis, conducted using G*Power 3, determined that a sample size of 36 participants was necessary to achieve a statistical power of 0.95. This was to detect median-sized effects (*f* = 0.25) with an alpha level of .05 in a two-factor within-participants repeated measures analysis of variance (ANOVA). The sample included 45 right-handed adults (24 females) aged 18–23 years (mean ± SD = 19.86 years ± 1.31 years). All had normal or corrected-to-normal vision, had no neurological or psychiatric disorders, had no acute or chronic pain, and were not using any medication. Participants were required to finish their meals 2 h before the experiment. Written informed consent was obtained from all individuals. This study conformed to the Declaration of Helsinki and was approved by the local research ethics committee of Chongqing Normal University. All procedures followed ethical guidelines and regulations.

#### Stimuli

##### Spicy and control treatments

**Figure 1.**
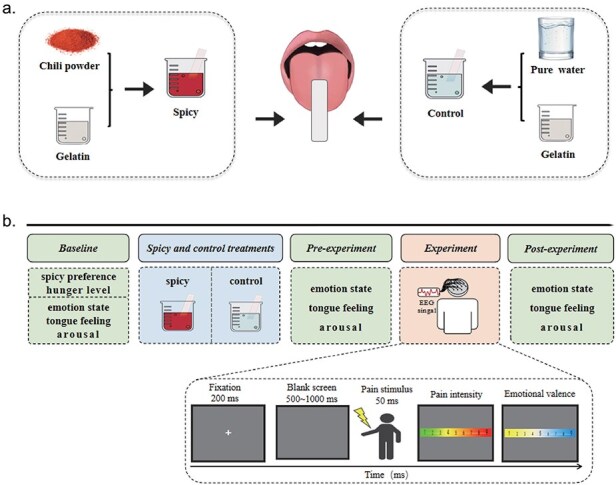
Experimental treatments and design of Study 2. (a) Spicy and control treatments. Spicy treatment: spicy solution was made by mixing chili powder with a gelatin base solution; Control treatment: control solution was made by mixing pure water with a gelatin base solution. (b) Flowchart describing the entire procedure of Study 2.

To ensure equivalent conditions between the two treatments in this study, a standardized method was employed for the formulation and application of the spicy and control treatments to participants based on a previous study (Duan et al. 2020). As shown in Fig. 1a, initially, 15 ml of 2% bovine bone gelatin (Bakerdream, China) was softened with cold water and then melted with heat to create a base gelatin solution. For the spicy treatment, this base gelatin solution was thoroughly mixed with 4 g of chili powder (Simply Organic, USA), resulting in the spicy solution. For the control treatment, the base gelatin solution was mixed with an equivalent volume of pure water, resulting in the control solution. Subsequently, filter paper (2 cm **×** 8 cm) was dipped in either the spicy solution or the control solution and placed on the participants’ tongue. Participants were then asked to hold the filter paper with the spicy (spicy treatment) solution or the control (control treatment) solution in their mouths for 1 min (Lee et al. 2009).

##### Pain stimuli

Pain stimuli were delivered through electrodes placed on the dorsum of the left hand using a constant current stimulator (SXC-4A, Sanxia Technique Inc., China). According to published studies (Hu et al. 2014, Boll et al. 2018, Peng et al. 2021, Wei et al. 2023, Yang et al. 2023), the calibrated electrical currents for stimuli in Study 2 were 1200 μA (minor pain) and 3800 μA (moderate pain) for female participants, 2755 μA (minor pain) and 4505 μA (moderate pain) for male participants.

#### Procedure

The experiment was conducted in a comfortable, quiet room with an ambient temperature of ∼25^°^C. All participants were seated ∼80 cm from a 24-inch computer screen. Stimuli presentation was controlled using the E-Prime (3.0) program (Psychology Software Tools, Inc., Pittsburgh, PA, USA).

Figure 1b presents the entire procedure of Study 2. Each participant participated in two treatments. Participants were randomly assigned to either the spicy treatment or the control treatment first, with the order counterbalanced among participants, and participated in the other treatment after a 1-week interval. Before each treatment, participants rated their current hunger level (1* =* ‘very hungry’, 9 = ‘very full’) and spicy preference (1 = ‘entirely dislike’, 9 = ‘entirely like’) using nine-point Likert scales to ensure equivalent physical conditions between the two treatments. No significant differences in hunger level or spicy preference were found between the treatments (all *P* > .05) using paired-samples *t*-test analyses (for details, see Appendix 1).

To ensure a persistent pungency in the mouth throughout the spicy treatment and monitor the physiological effects of both treatments, participants assessed their current state (State Assessment) three times: before the treatment (baseline), before the experiment (pre-experiment), and after the experiment (post-experiment). These assessments included tongue feeling (0* =* ‘no sensation’, 4 = ‘pain threshold’, 10 = ‘utmost pain imaginable’) (Bijur et al. 2001), emotional state (1* =* ‘very unhappy’, 9 = ‘very happy’), and arousal (1 = ‘extremely peaceful’, 9 = ‘extremely excited’).

Prior to the experiment, participants completed a training session to get acquainted with the procedure. Each trial of the experiment for electroencephalogram (EEG) recording began with a 200-ms fixation cross on a grey screen, followed by a blank screen for 500 to 1000 ms, and then a 50-ms pain stimulus was presented. Participants were instructed to respond as accurately and quickly as possible with a key-press (either ‘1’ or ‘2’) to indicate whether the stimulus was minor pain or moderate pain, with key-press order counterbalanced among participants. They then rated the pain intensity (1* =* ‘no sensation’, 4 = ‘pain threshold’, 9 = ‘unbearable pain’) and emotional valence (1* =* ‘very unhappy’, 9 = ‘very happy’) for each stimulus according to the 9-point Likert scale. The inter-trial interval ranged from 4000 to 6000 ms. The study included 40 trials for each treatment, totalling 80 trials.

#### EEG recording and data analyses

EEG data were recorded from 64 scalp sites using tin electrodes mounted on an actiCHamp system (Brain Vision LLC, Morrisville, NC, USA; pass band: 0.01∼100 Hz; sampling rate: 1000 Hz). The FCz electrode served as the recording reference, with an electrode on the medial frontal aspect. All electrode impedances remained <5 kΩ.

EEG data were preprocessed and analysed via MATLAB R2016a (MathWorks, Natick, MA, USA) and the EEGLAB toolbox (Delorme and Makeig 2004). Continuous EEG signals were band-passed filtered (0.1–30 Hz) and segmented using a 1000-ms time window. Time windows of 200 ms before and 800 ms after the onset of pain stimuli were extracted from the continuous EEG. EEG epochs were baseline-corrected by a 200-ms interval prior to the pain stimuli onset. Electro-oculographic artefacts were corrected with an independent component analysis algorithm (Jung et al. 2001).

After confirming scalp topographies in both single-participant and group-level ERP waveforms, and based on published research (Meng et al. 2013, Hu et al. 2014, Li et al. 2022, 2024, Yang et al. 2023), the dominant ERP components, including N1 and P2, were identified. These components are strongly associated with the neural processing of pain stimuli (Kakigi et al. 2000, Tiemann et al. 2018, Zhuo et al. 2023).

Amplitudes and latencies of N1 were measured at right-central electrodes (Fz, F2, F4, FCz, FC2, FC4, Cz, C2, and C4) contralateral to the side of pain stimuli, with average N1 amplitudes analysed within latency intervals of 80–120 ms. Amplitudes and latencies of P2 were measured at the central electrodes (FC1, FCz, FC2, C1, Cz, C2, CP1, CPz, and CP2), with average P2 amplitudes analysed within latency intervals of 190–230 ms.

#### Statistical analyses

Data analyses were performed using MATLAB R2016a (MathWorks, Natick, MA, USA). The State Assessments of tongue feeling, emotional state, and arousal were explored using a repeated measures ANOVA with two within-participant factors: ‘treatment’ (spicy treatment and control treatment) and ‘timepoint’ (baseline, pre-experiment, and post-experiment). If interactions between the two factors were significant, simple effects analyses between the three timepoints were implemented for each treatment.

Behavioural data (reaction time, accuracy, pain intensity, and emotional valence) and ERP data (N1 and P2 amplitude and latency) were analysed using a repeated measures ANOVA with two within-participant factors: ‘treatment’ (spicy treatment and control treatment) and ‘pain’ (minor pain and moderate pain). If interactions between the two factors were significant, simple effects analyses between the two treatments were performed for minor pain and moderate pain separately.

Additionally, to better understand the interactions of ‘treatment’ (spicy treatment and control treatment) and ‘pain’ (minor pain and moderate pain), the differential ERP waves between spicy and control treatments (e.g. the differential P2 between spicy and control treatments represented as P2_spicy-control_) for minor-pain and moderate-pain stimuli were calculated separately. Paired-samples *t*-tests were then used to compare the differential ERP amplitudes between minor-pain and moderate-pain stimuli.

## Results

### Study 1: The relationship between spicy food craving and pain perception

#### Common method bias test

In Study 1, all variables were derived from self-reported data provided by undergraduate students, which raises the potential issue of common method bias. To address this concern, we used Harman’s single-factor test (Podsakoff et al. 2003). The results indicated that the variance accounted for by the first principal factor was merely 25.45%, significantly below the 40% threshold. Thus, the risk of common method bias was negligible (Podsakoff et al. 2003).

#### Descriptive and correlation analysis

**Figure 2.**
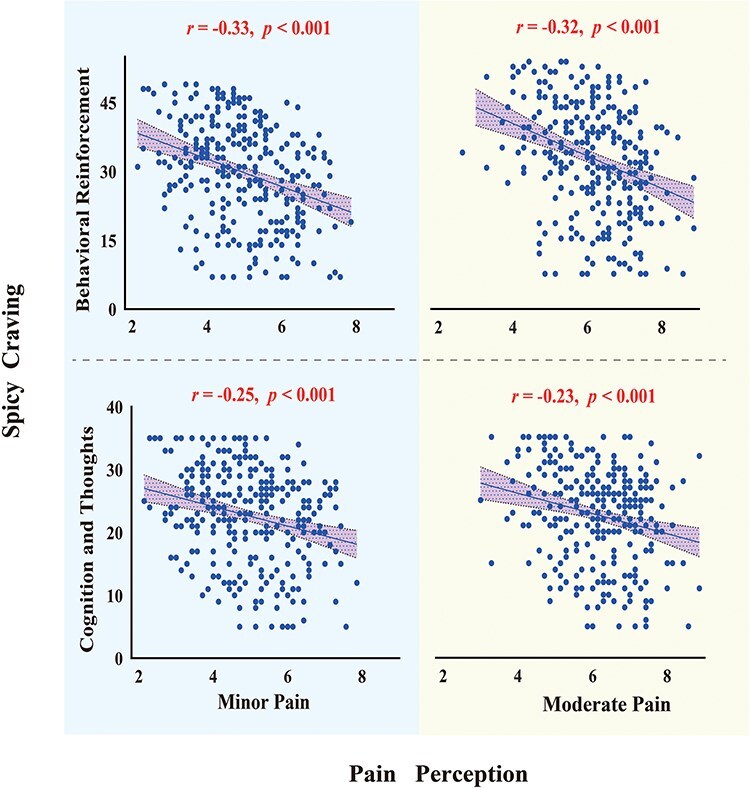
The relationship between subscales of Spicy food Craving and Pain Perception. Correlation between behavioural reinforcement and minor pain (top left panel), behavioural reinforcement and moderate pain (top right panel), cognition and thoughts and minor pain (bottom left panel), cognition and thoughts and moderate pain (bottom right panel).

The results suggested that Spicy Food Craving was negatively correlated with Pain Perception (*r* = −0.34, *P* < .001). Means, SDs, and Pearson’s correlation coefficients of the variables are shown in Table 1 and Fig. 2.

**Table 1.** Descriptive statistics and correlations among variables.

	*M *± SD	1	1.1	1.2	2	2.1
1. Spicy food craving	55.52 ± 17.48	–				
1.1. Behavioural reinforcement	29.86 ± 10.92	0.96^***^	–			
1.2. Cognition and thoughts	22.65 ± 7.59	0.92^***^	0.78^***^	–		
2. Pain perception	5.55 ± 1.03	−0.34^***^	−0.36^***^	−0.26^***^	–	
2.1 Minor pain	4.95 ± 1.19	−0.31^***^	−0.33^***^	−0.25^***^	0.91^***^	–
2.2 Moderate pain	6.14 ± 1.09	−0.30^***^	−0.32^***^	−0.23^***^	0.90^***^	0.64^***^

*Notes*: ^***^P < .001 Spicy food craving: scores of the Spicy Food Craving Questionnaire; behavioural reinforcement: scores of the behavioural reinforcement subscale of the Spicy Food Craving Questionnaire; cognition and thoughts: scores of the cognition and thoughts subscale of the Spicy Food Craving Questionnaire; pain perception: scores of the Pain Sensitivity Questionnaire; minor pain: scores of the minor pain subscale of Pain Sensitivity Questionnaire; moderate pain: scores of the moderate-pain subscale of the Pain Sensitivity Questionnaire.

### Study 2: The influence of spicy food intake on pain perception

#### Results of the State Assessment

**Figure 3.**
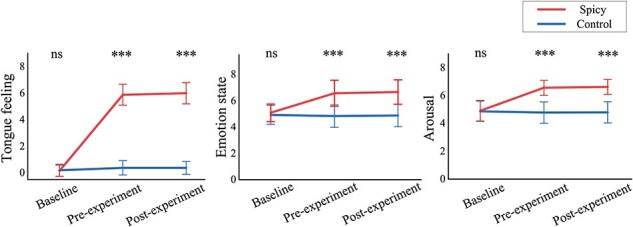
Statistics analysis results of the State Assessments. Line charts describing results of State Assessments in the spicy and control treatments. Tongue feeling (left panel), emotional state (middle panel), and arousal (right panel) were assessed before treatment (baseline), before the experiment (pre-experiment), and after the experiment (post-experiment). Data are expressed as *M* ± SD. ns: *P* > .05, ^***^: *P* < .001.

The State Assessments of tongue feeling, emotional state, and arousal were analysed at three timepoints: baseline, pre-experiment, and post-experiment. The details of the State Assessments are presented in Fig. 3. For the spicy treatment, tongue feeling and arousal were more intense, and emotional state was more positive at pre-experiment and post-experiment assessments than at baseline (all *P* < .001). No significant differences were found between pre-experiment and post-experiment assessments, nor among the three timepoints for the control treatments (all *P* > .05) (for details, see Appendix 2).

#### Behavioural results

##### Reaction time

Reaction times were modulated by the main effects of ‘treatment’ (*F*_(1,44)_ = 12.06, *P* = .001, η_p_^2^ = 0.22) and ‘pain’ (*F*_(1,44)_ = 10.16, *P* = .003, η_p_^2^ = 0.19). Participants responded more quickly to the pain stimuli after spicy treatment (478.96 ± 17.78 ms) than after control treatment (540.63 ± 19.55 ms), and more quickly to minor-pain stimuli (485.84 ± 18.17 ms) than to moderate-pain stimuli (533.74 ± 17.98 ms).

##### Accuracy

Accuracies were modulated by the main effects of ‘treatment’ (*F*_(1,44)_ = 13.21, *P* < .001, η_p_^2^ = 0.23) and ‘pain’ (*F*_(1,44)_ = 12.99, *P* < .001, η_p_^2^ = 0.23). Participants judged the stimuli less accurately after spicy treatment (74.21% ± 2.90%) than after control treatment (83.64% ± 2.01%), and responded less accurately to minor-pain stimuli (74.82% ± 2.52%) than to moderate-pain stimuli (83.13% ± 2.30%).

##### Pain intensity

Pain intensity ratings were modulated by the main effects of ‘treatment’ (*F*_(1,44)_ = 191.22, *P* < .001, η_p_^2^ = 0.81) and ‘pain’ (*F*_(1,44)_ = 562.36, *P* < .001, η_p_^2^ = 0.93). Participants felt less pain from the pain stimuli after spicy treatment (5.48 ± 0.10) than after control treatment (6.59 ± 0.09), and minor-pain stimuli (5.12 ± 0.10) were judged as less painful than moderate-pain stimuli (6.95 ± 0.09). Pain intensity ratings were also modulated by the interaction between ‘treatment’ and ‘pain’ (*F*_(1,44)_ = 4.84, *P* = .033, η_p_^2^ = 0.10). The differences between spicy and control treatments were larger for minor-pain stimuli (spicy treatment: 4.48 ± 0.11, control treatment: 5.77 ± 0.13; *P* < .001) than for moderate-pain stimuli (spicy treatment: 6.47 ± 0.12, control treatment: 7.42 ± 0.09; *P* < .001).

##### Emotional valence

Emotional valence ratings were modulated by the main effects of ‘treatment’ (*F*_(1,44)_ = 47.02, *P* < .001, η_p_^2^ = 0.52) and ‘pain’ (*F*_(1,44)_ = 148.67, *P* < .001, η_p_^2^ = 0.77). Participants felt less negative about the pain stimuli after spicy treatment (4.94 ± 0.10) than after control treatment (4.17 ± 0.10), and minor-pain stimuli (5.36 ± 0.10) were judged less negative than moderate-pain stimuli (3.76 ± 0.11). Emotional valence ratings were also modulated by the interaction between ‘treatment’ and ‘pain’ (*F*_(1,44)_ = 11.22, *P* = .002, η_p_^2^ = 0.20). The differences between spicy and control treatments were larger for minor-pain stimuli (spicy treatment: 5.90 ± 0.14, control treatment: 4.81 ± 0.10; *P* < .001) than for moderate-pain stimuli (spicy treatment: 3.99 ± 0.13, control treatment: 3.54 ± 0.14; *P* = .003).

#### ERP results

**Figure 4.**
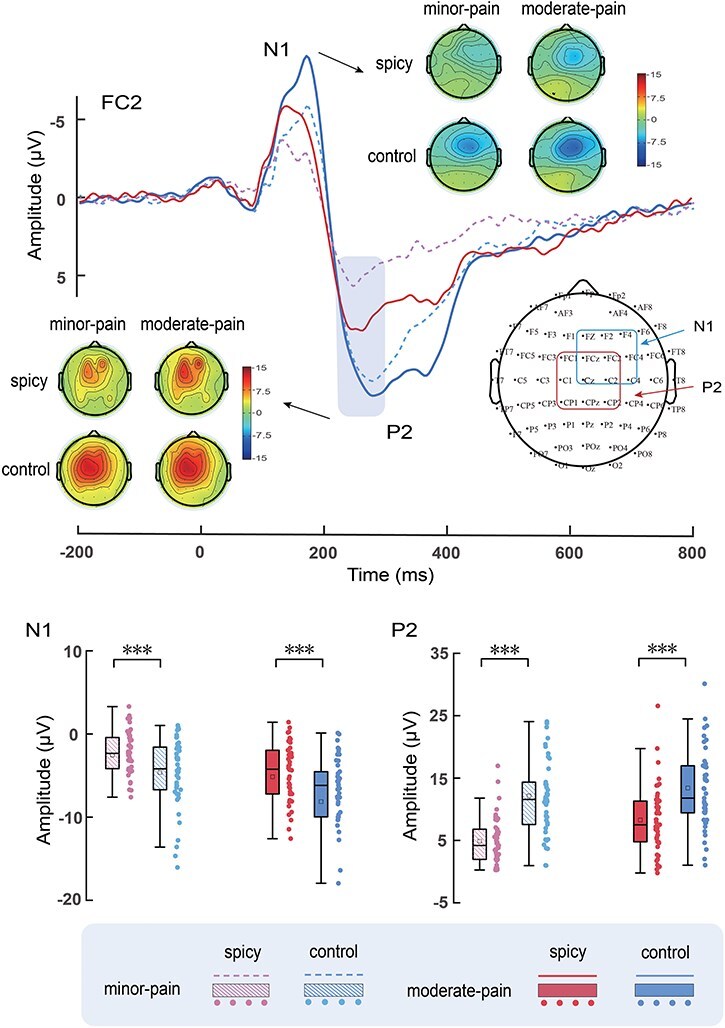
ERP waveforms, scalp topography distributions, and bar charts. ERP waveforms and scalp topography distributions exhibited by participants in response to minor-pain (dotted) and moderate-pain (solid) stimuli after spicy and control treatments. Electrodes used to estimate ERP amplitudes are marked using squares on the topographic distributions. Data in the bar charts are expressed as mean ± SEM. ^***^P < .001.

ERP waveforms, scalp topographies, and bar charts for Study 2 are shown in Fig. 4. Results of the statistical analyses of ERP amplitudes are summarized in Table 2.

**Table 2.** Results of the statistical analyses of the ERP amplitudes.

	N1			P2		
Variable	*F*	*P*	η_p_^2^	*F*	*P*	η_p_^2^
Treatment	**28.74**	**<.001**	**0.40**	**59.65**	**<.001**	**0.58**
Pain	**33.88**	**<.001**	**0.44**	**22.90**	**<.001**	**0.34**
Treatment × Pain	2.44	.126	0.05	**8.76**	**.005**	**0.17**

*Notes*: Results were obtained using repeated-measures ANOVA with the within-participant factors of ‘treatment’ (spicy treatment and control treatment) and ‘pain’ (minor pain and moderate pain). Significant comparisons (*P* < .05) are indicated in boldface.

#### ERP amplitude

##### N1

N1 amplitudes were modulated by ‘treatment’ (*F*_(1,44)_ = 28.74, *P* < .001, η_p_^2^ = 0.40) and ‘pain’ (*F*_(1,44)_ = 33.88, *P* < .001, η_p_^2^ = 0.44), the spicy treatment (−3.84 ± 0.66 μV) elicited decreased N1 amplitudes compared to the control treatment (−6.38 ± 0.84 μV), and minor-pain stimuli (−3.58 ± 0.54 μV) elicited decreased N1 amplitudes compared to moderate-pain stimuli (−6.64 ± 0.94 μV).

##### P2

P2 amplitudes were modulated by the main effects of ‘treatment’ (*F*_(1,44)_ = 59.65, *P* < .001, η_p_^2^ = 0.58) and ‘pain’ (*F*_(1,44)_ = 22.90, *P* < .001, η_p_^2^ = 0.34), the spicy treatment (6.57 ± 0.62 μV) elicited smaller P2 amplitudes than the control treatment (12.78 ± 0.99 μV), and minor-pain stimuli (8.51 ± 0.74 μV) elicited smaller amplitudes than moderate-pain stimuli (10.84 ± 0.78 μV). P2 amplitudes were also modulated by the interaction between ‘treatment’ and ‘pain’ (*F*_(1,44)_ = 8.76, *P* = .005, η_p_^2^ = 0.17). The differences between spicy and control treatments tended to be larger for minor-pain stimuli (spicy treatment: 4.87 ± 0.57 μV, control treatment: 12.15 ± 1.06 μV; *P* < .001) than for moderate-pain stimuli (spicy treatment: 8.27 ± 0.80 μV, control treatment: 13.41 ± 1.01 μV; *P* < .001).

##### Differential ERP amplitude

**Figure 5.**
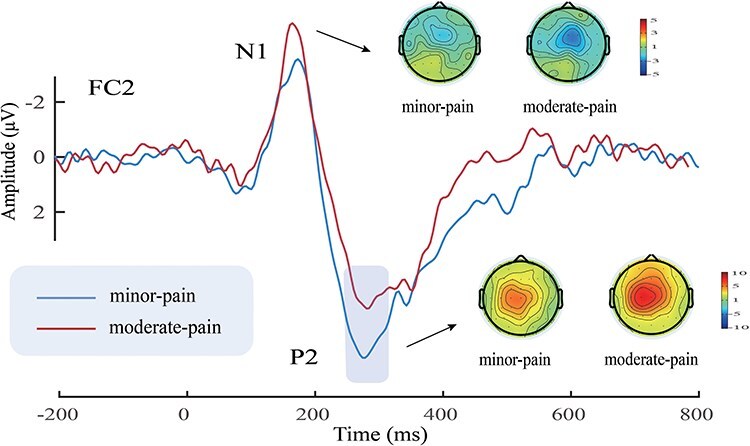
Waveforms and topographic maps of the differential ERP waves between spicy and control treatments. The lines respectively represent the differential ERP waveform (spicy-control) for moderate-pain stimuli and the differential ERP waveform (spicy-control) for minor-pain stimuli.

Waveform and topographic maps of the differential ERP waves between spicy and control treatments are shown in Fig. 5. The differential ERP amplitudes between spicy and control treatments for P2_spicy-control_ were larger for minor-pain stimuli (7.27 ± 0.83 μV) than for moderate-pain stimuli (5.14 ± 0.93 μV) (*t*_(44)_ = 2.96, *P* = .005, Cohen’s *d* = 0.44). However, the differential ERP amplitudes for N1_spicy-control_ were not significantly different between minor-pain (−2.09 ± 0.46 μV) and moderate-pain stimuli (−2.98 ± 0.63 μV) (*t*_(44)_ = 1.56, *P* = .126, Cohen’s *d* = 0.23).

#### ERP latency

##### N1

N1 latencies were modulated by ‘treatment’ (*F*_(1,44)_ = 30.13, *P* < .001, η_p_^2^ = 0.41), with spicy treatment (97.09 ± 1.49 ms) eliciting shorter N1 latencies than control treatment (105.05 ± 1.83 ms). No other significant main effects or interaction for N1 latency were found (*P* > .05).

##### P2

P2 latencies were modulated by ‘treatment’ (*F*_(1,44)_ = 33.70, *P* < .001, η_p_^2^ = 0.43), with spicy treatment (208.69 ± 1.45 ms) eliciting shorter P2 latencies than control treatment (217.55 ± 1.55 ms). No other significant main effects or interactions for P2 latency were found (*P* > .05).

#### Correlation between the behavioural data and the ERP data

The N1 amplitudes were positively correlated with the pain intensity ratings (*r* = 0.39, *P* = .009) when receiving minor-pain stimulation under control treatment conditions. The P2 amplitudes were negatively correlated with the pain intensity ratings (*r* = −0.34, *P* = .023) when receiving moderate-pain stimulation under control treatment conditions. No other significant correlation was found between the behavioural data and the ERP data (for details, see Table C2 of Appendix 3).

## Discussion

The present study aimed to investigate the analgesic effect of spicy food intake. Study 1 explored the relationship between individuals’ craving for spicy food and their pain perception using questionnaires, finding a negative correlation. Study 2 used the ERP experimental method to explore the behavioural-neural responses to pain stimuli following spicy and control treatments. The results showed that, compared to control treatments, spicy treatments resulted in shorter reaction times, lower accuracies and pain intensity ratings, less negative emotional responses, smaller N1 and P2 amplitudes, and shorter N1 and P2 latencies in response to pain stimuli, especially minor-pain stimuli.

### Study 1: Spicy food craving negatively correlated with pain perception

Study 1 found a negative relationship between Spicy Food Craving and Pain Perception, suggesting that individuals who enjoy spicy food intend to have lower pain perception. Significant negative correlations were also found between Spicy Food Craving and two subscales of the Pain Sensitivity Questionnaire: the minor-pain subscale (indicating minor-pain perception) and the moderate-pain subscale (indicating moderate-pain perception). As correlation results do not establish causation, Study 2 was designed to further explore the impact of spicy food intake on pain perception and its underlying neural mechanisms by setting up two intensities of pain stimuli: minor pain and moderate pain.

Additionally, we also found that Pain Perception was negatively correlated with two subscales of the Spicy Food Craving Questionnaire: the behavioural reinforcement subscale (indicating behaviour and positive reinforcement for eating spicy food) and the cognition and thoughts subscale (indicating thoughts, cravings, and intentions for spicy food).

### Study 2: Spicy food intake decreased pain perception

The main effects of ‘pain’ were found in both behavioural and ERP results in Study 2. Consistent with previous studies (Meng et al. 2013, Peng et al. 2019, Zhang et al. 2022, Wang et al. 2023, Wei et al. 2023, Yang et al. 2023), enhanced pain intensity ratings, more negative emotional reactions, and larger N1 and P2 amplitudes to moderate-pain stimuli than minor-pain stimuli were found in Study 2. Additionally, on the pain intensity rating scale (1* = *no sensation, 4 = pain threshold, 9 = unbearable pain), the mean ratings for minor-pain and moderate-pain stimuli were 5.12 and 6.95, respectively, indicating that the chosen pain stimuli were appropriate for this study.

The main effects of ‘treatment’ were also observed in both behavioural and ERP results in Study 2. Lower pain intensity ratings, less negative emotional responses, and smaller N1 and P2 amplitudes were elicited after spicy treatment than control treatment, indicating that spicy food intake can decrease pain perception. These findings align with a previous study using spicy and nonspicy food pictures as priming stimuli (Yang et al. 2023). Given that the N1 and P2 components represent early sensory processing (Lee et al. 2009, Mouraux and Iannetti 2009, Peng et al. 2019, Zhang et al. 2023) and subsequent affective-motivational processing (Rütgen et al. 2015, Li et al. 2023) of pain and are sensitive to attention allocation (Hillyard et al. 1973, Luck and Hillyard 1994, Dong et al. 2024), spicy food may reduce attention to pain stimuli by eliciting mild discomfort. This distracting effect may decrease the sensory and affective-motivational processing of pain stimuli.

Interestingly, P2 amplitudes were modulated by the interaction between ‘treatment’ and ‘pain’. Although the P2 amplitudes for both minor-pain and moderate-pain stimuli were reduced more after spicy treatment than after control treatment, the differential P2 amplitudes between spicy and control treatments were larger for minor-pain stimuli than for moderate-pain stimuli. These findings suggest that the analgesic effect of spicy food on pain perception may vary with the intensity of the pain stimuli and that this effect may be more pronounced for minor-pain stimuli.

There was also a correlation between behavioural data and ERP data. N1 amplitudes were significantly correlated with the pain intensity ratings when receiving minor-pain stimulation under control treatment conditions, and P2 amplitudes were significantly correlated with the pain intensity ratings when receiving moderate-pain stimulation under control treatment conditions. However, there was no significant correlation between pain intensity ratings and the N1 and P2 amplitudes in the spicy treatment conditions. These results suggest that the intake of spicy foods may affect the processing of pain.

#### The analgesic effect of spicy food intake on pain perception

The present study suggests that spicy food intake may have an analgesic effect. One possible explanation for this phenomenon may be related to the release of endorphins. Research indicated that subcutaneous capsaicin injection could elevate beta-endorphin concentration in cerebrospinal fluid (Gear et al. 1999, Duan et al. 2020, Aghaei and Ebrahimi Moghaddam 2024), alleviating pain and generating feelings of pleasure.

Another explanation for the antinociceptive effect of spicy food stimulation could be its connection to intrinsic reward-motivation mechanisms (Gourisankar et al. 2018, Wang et al. 2020). When individuals are subjected to pain stimuli, they may shift their attention from their personal suffering to the intrinsic reward-motivation mechanisms (Gear et al. 1999, Meule et al. 2013, Yang et al. 2023), resulting in a positive affective-emotional experience that helps alleviate the perception of pain (Navratilova et al. 2016).

Lastly, the analgesic effects of spicy stimulation may be attributed to the modulation of pain mechanisms. Capsaicin, the pungent ingredient in spicy food, induces pain by activating the TRPV1 receptor (Bode and Dong 2011, Choy et al. 2021), while also triggering conditioned pain modulation (CPM), an endogenous pain inhibitory process triggered by pain stimuli in humans in a specific temporal pattern (King 2014). CPM is typically tested in the lab using a variety of ‘pain-inhibits-pain’ paradigms by two remote noxious stimuli: one, the ‘conditioning stimulus’, inhibiting the other, the ‘test-stimulus’. In this study, the pungent sensation was the ‘conditioned stimulus’ and the electric pain stimulus was the ‘test-stimulus’ (Nirl et al. 2011). CPM is seen as psychophysical equivalent to the physiological mechanism ‘diffuse noxious inhibitory controls’, which explains the effectiveness of the ‘pain-inhibits-pain’ counter-irritation. Via spino-bulbo-spinal loops, activity of pain-signalling neurons in the spinal dorsal horn and in trigeminal nuclei is attenuated in response to noxious stimuli applied to a remote area of the body (Moont et al. 2010, Horn‐Hofmann et al. 2024). Emotional states also significantly affect the body’s ability to modulate pain, with more positive emotions enhancing pain inhibition (Lang-Illievich et al. 2024). For the spicy treatment, tongue feeling and arousal were more intense, and emotional state was more positive at pre-experiment and post-experiment assessments than at baseline. However, there was no significant correlation between emotional state after spicy treatment and ERP amplitude, pain intensity score, or emotional valence (for details, see Table C1 of Appendix 3), suggesting that pain relief from spicy food intake is more likely to occur through CPM. Subdermal capsaicin injection has been found to produce antinociceptive effects by activating opioid receptors in the central nucleus accumbens (Gear et al. 1999, Arora et al. 2021, 2022).

Despite the methodological rigour of these studies, several potential limitations should be considered. First, the participants recruited were healthy adults, so it is not clear whether the results can be replicated in other populations, especially those with chronic pain or medical conditions. Secondly, the ERP technique used did not identify specific brain activation regions, which could be addressed in future research through the application of fMRI. Thirdly, due to the scope and design limitations of this study, we were unable to include variables such as food preferences, personality, emotional traits and status, and HPA measurements. Combining these variables may result in a more comprehensive model and provide more convincing explanations, which we hope to address in future studies. Furthermore, the generalizability of the findings may be influenced by the local food culture as the study was conducted in Chongqing, a city renowned for its spicy cuisine. It is important to consider that individuals in this region might have distinct experiences or perceptions regarding spicy food compared to those residing in less prevalent or popular regions. Finally, while our study demonstrated significant analgesic effects of spicy food intake in alleviating acute pain, the focus was limited to minor- and moderate-pain levels. Considering that spicy food intake may also cause negative effects such as inflammation, the use of spicy food as a pain relief strategy needs further research. Further investigation is needed to explore these possibilities. In the future, it is also possible to analyze patients’ pain-related data with the help of artificial intelligence algorithms and establish personalized pain models (Wang et al. 2024, Kuai et al. 2021, 2024, 2025). By using artificial intelligence technology to analyze a large amount of biomedical data, researchers can identify the possible analgesic targets of capsaicin quickly and accurately (Khondakar et al. 2024, Vimbi et al. 2024).

In summary, the present research used two studies to explore the analgesic effect of spicy food intake on pain perception. Study 1 found a negative correlation between spicy food craving and pain perception in daily life using a questionnaire method. Study 2 demonstrated that spicy food intake can effectively decrease pain perception using ERP techniques. Together, these findings revealed the analgesic effects of eating spicy food, potentially offering a novel perspective on dietary interventions for pain.

## Appendix 1 Statistical results of hunger levels and spicy preference

The result of paired-samples *t*-test revealed that hunger levels of the participants did not differ significantly (*t*_(44)_ = 0.17, *P* = .864) between the spicy (5.22 ± 0.97) and control (5.20 ± 1.32) treatments, indicating that there was essentially no difference in the hunger levels of participants who came to the experiment on two separate occasions and both remained moderately full before the two treatments.

The result of paired-samples *t*-test suggested that spicy preferences of the participants was no significant differences(*t*_(44)_ = 1.57, *P* = .124) between the spicy (6.11 ± 2.37) and control (5.89 ± 1.99) treatments, showing that individuals’ preferences for spiciness exhibit a certain degree of stability, with participants demonstrating essentially the same preference for spicy food across two experimental sessions.

## Appendix 2 Statistical results of the State Assessments

The main effects of ‘treatment’ were significant for the tongue feeling ratings (*F*_(1,44)_ = 2220.52, *P *< .001, η_p_^2^ = 0.98), emotional states (*F*_(1,44)_ = 78.29, *P* < .001, η_p_^2^ = 0.64), and arousal (*F*_(1,44)_ = 116.20, *P* < .001, η_p_^2^ = 0.73), the tongue feeling and arousal of spicy treatment were more intense than control treatment, the emotional states of spicy treatment were more positive than control treatment.

The main effects of ‘timepoint’ were significant for the tongue feeling ratings (*F*_(1,43)_ = 909.40, *P* < .001, η_p_^2^ = 0.95), emotional states (*F*_(1,43)_= 51.88, *P* < .001, η_p_^2^ = 0.54), and arousal (*F*_(1,43)_ = 56.75, *P* < .001, η_p_^2^ = 0.56). This indicated that for both tongue feeling and arousal, pre-experiment and post-experiment assessments were more intense than baseline assessment (all *P* < .001). Moreover, emotional state ratings of both pre-experiment and post-experiment assessments were more positive than those of baseline assessment (all *P* < .001).

In addition, the tongue feeling (*F*_(1,43)_ = 657.94, *P* < .001, η_p_^2^ = 0.94), emotional states (*F*_(1,43)_ = 50.35, *P* < .001, η_p_^2^ = 0.53), and arousal (*F*_(1,43)_ = 104.82, *P* < .001, η_p_^2^ = 0.70) were modulated by the interaction between ‘treatment’ and ‘timepoint’. The simple effects analysis indicated that, for spicy treatment, tongue feeling and arousal were more intense, and emotional state were more positive at pre-experiment and post-experiment assessments than at baseline assessment (all *P* < .001), while no difference was found between pre-experiment and post-experiment assessment, nor the three timepoint for control treatments (*P* > .05).

## Appendix 3

**Table C1.** Correlation analysis results of spicy food craving, emotional state, pain intensity ratings, and emotional valence.

	1	2	3	4	5	6	7	8	9
Spicy food craving									
Emotional state after spicy treatment	0.07								
Pain intensity ratings when receiving minor-pain stimulation under spicy treatment conditions	−0.03	0.14							
Pain intensity ratings when receiving moderate-pain stimulation under spicy treatment conditions	0.05	<−0.01	0.62^***^						
Pain intensity ratings when receiving minor-pain stimulation under control treatment conditions	0.05	0.11	0.48^***^	0.54^***^					
Pain intensity ratings when receiving moderate-pain stimulation under control treatment conditions	0.11	0.03	0.49^***^	0.57^***^	0.46^***^				
Emotional valence when receiving minor-pain stimulation under spicy treatment conditions	−0.10	0.01	0.04	−0.06	−0.05	−0.02			
Emotional valence when receiving moderate-pain stimulation under spicy treatment conditions	0.16	0.23	0.09	0.15	−0.17	0.09	0.09		
Emotional valence when receiving minor-pain stimulation under control treatment conditions	−0.05	−0.11	0.06	−0.08	0.05	0.25	0.23	−0.05	
Emotional valence when receiving moderate-pain stimulation under control treatment conditions	0.12	0.12	−0.20	−0.06	−0.28	−0.01	0.20	0.45^**^	0.37^*^

Note: *: p < 0.05, **: p < 0.01, ***: p < 0.001.

**Table C2.** Correlation analysis results of spicy food craving, emotional state, and ERP amplitude.

	1	2	3	4	5	6	7	8	9
Spicy food craving									
Emotional state after spicy treatment	0.07								
N1 amplitude when receiving minor-pain stimulation under spicy treatment conditions.	−0.03	−0.11							
N1 amplitude when receiving moderate-pain stimulation under spicy treatment conditions	−0.02	0.05	0.84^***^						
N1 amplitude when receiving minor-pain stimulation under control treatment conditions	<−0.01	0.01	0.69^***^	0.64^***^					
N1 amplitude when receiving moderate-pain stimulation under control treatment conditions	−0.01	−0.02	0.81^***^	0.84^***^	0.84^***^				
P2 amplitude when receiving minor-pain stimulation under spicy treatment conditions	0.14	0.14	0.13	0.15	0.15	0.17			
P2 amplitude when receiving moderate-pain stimulation under spicy treatment conditions	0.22	0.08	0.12	0.13	0.03	0.15	0.62^***^		
P2 amplitude when receiving minor-pain stimulation under control treatment conditions	0.02	−0.01	−0.07	0.07	−0.03	−0.02	0.62^***^	0.35^*^	
P2 amplitude when receiving moderate-pain stimulation under control treatment conditions	0.03	0.02	<−0.01	0.11	<−0.01	0.06	0.65^***^	0.50^***^	0.85^***^

Note: *: p < 0.05, **: p < 0.01, ***: p < 0.001.
